# Making infection prevention and control everyone's business? Hospital staff views on patient involvement

**DOI:** 10.1111/hex.12874

**Published:** 2019-02-17

**Authors:** Elizabeth Sutton, Liz Brewster, Carolyn Tarrant

**Affiliations:** ^1^ Health Sciences University of Leicester Leicester UK; ^2^ University of Lancaster Lancaster UK

**Keywords:** co‐production, hand hygiene, infection prevention and control, patient involvement, patient safety

## Abstract

**Context:**

Ensuring an infection‐free environment is increasingly seen as requiring the contribution of staff, patients and visitors. There is limited evidence, however, about how staff feel about collaborating with patients and relatives to co‐produce that environment.

**Aims:**

This study aims to understand how hospital staff perceive the involvement of patients and relatives in infection prevention and control (IPC) and the main challenges for staff in working together with patients and relatives to reduce the threat of infection.

**Methods:**

Qualitative semi‐structured interviews were conducted with 35 frontline health‐care professionals and four executive staff, from two hospital trusts.

**Findings:**

We found that staff were more supportive of approaches that encourage co‐operation from patients and relatives, than of interventions that invoked confrontation. We identified challenges to involvement arising from staff concerns about shifting responsibility for IPC onto patients. Staff were not always able to work with patients to control infection risks as some patients themselves created and perpetuated those risks.

**Conclusions:**

Our work highlights that IPC has particular features that impact on the possibilities for involving patients and relatives at the point of care. Staff acknowledge tensions between the drive to involve patients and respect their autonomy, and their duty to protect patients from risk of unseen harm. The role that patients and relatives can play in IPC is fluctuating and context dependent. Staff responsibility for protecting patients from the risk of infection may sometimes need to take priority over prerogatives to involve patients and relatives in the co‐production of IPC.

## 1 INTRODUCTION

The much‐repeated mantra that infection prevention and control (IPC) is “everyone's business”^1^ is frequently understood to include not only staff of all roles and grades, but also patients and their relatives, all of whom are seen as stakeholders with a part to play in achieving IPC goals. There is limited evidence, however, about how staff feel about working together with patients and relatives to co‐produce that environment, or the specific challenges that staff perceive in relation to involving patients in IPC.

Co‐production in health care has patient involvement at its heart, recognizing the equal importance of both professional and patient knowledge in coming together to ensure that the service meets the needs of the user, and to improve the quality of care provided.^2^, ^3^, ^4^ Co‐production has its origins in two main camps—(a) the democratic ideal that emphasizes citizen rights and the “co‐production of public good” and (b) the shift away from viewing patients as passive recipients of health care to more informed engaged participants in the health‐care process.^3^ While co‐production can happen at various points across the design, delivery and evaluation of care, most co‐production activities targeting health‐care improvement take place away from the bedside, and consist of specific improvement projects in dedicated group‐work spaces.^2^, ^5^ Existing approaches to co‐production have attracted criticism for potentially conceptualizing the service users contribution as an “add on” to improving services, rather than seeing service users as integral at the outset.^6^ Knowing when and how to involve patients and relatives meaningfully in the joint endeavour of preventing infection is complex.

### 1.1 Patients’ roles in IPC

Patients have been identified as having a potentially important role in monitoring and encouraging good IPC practices,^7^, ^8^, ^9^, ^10^ with some studies reporting, for example, that involving patients as observers of hand hygiene practices improves clinician adherence.^11^, ^12^ Despite this, evidence suggests that staff and patients have some reservations about patient involvement in IPC, particularly where this comprises patients challenging staff about their practices.^11^, ^12^ While many patients are, in theory, willing to ask staff to wash their hands, they are often unwilling to do so in practice.^7^, ^13^, ^14^, ^15^, ^16^ Patients and relatives report barriers to acting as “vigilant monitors”^17^ by monitoring their care and speaking up about concerns: including fear of the consequences of raising concerns; a respect of medical authority that makes them reluctant to question staff about their behaviour^18^; and a desire to avoid disturbing busy nursing staff by asking questions.^19^ Patients may also lack volition^17^ to be involved in IPC due to feeling ill or vulnerable, or feeling that they lack the skills or knowledge. Evidence suggests that patients have limited understanding of infection risks and this may be a significant barrier to playing an active part in efforts to reduce these risks.^13^, ^20^, ^21^

If patients are to be involved in IPC, then frontline health‐care staff need to be engaged and empowered to support patient involvement.^21^, ^22^ Staff efforts to support and encourage patients to play a role in IPC can break down barriers and reassure patients that they have an important role to play.^16^ The way that patient and relative feedback is received and managed by staff is a critical factor influencing how comfortable patients feel about speaking up,^23^ but staff may find being questioned by patients difficult to handle.^24^ While there have been a number of studies of patients’ views on their involvement in safety and IPC, little is known about staff views and attitudes towards patient and relative involvement in IPC.

This article is drawn from research that identified and synthesized the evidence from infection prevention initiatives with qualitative case studies in two hospital NHS trusts.^25^ From this, our article aims to understand how hospital staff perceive the involvement of patients and relatives in the co‐production of IPC in practice, and to highlight the main challenges that impact on collaborating with patients and relatives to reduce the threat of infection on hospital wards.

## 2 METHODS

Semi‐structured interviews were conducted with nursing and support staff purposively sampled from two hospital trusts in England, and staff with management and executive responsibilities including hospital‐wide responsibility for IPC. Participants (n = 29) were initially recruited from six medical and surgical wards in one trust. In order to provide a wider range of hospital environments, a further ten participants were recruited from intensive care and accident and emergency in the second trust. One trust was a teaching hospital NHS trust and one a university hospital foundation trust. Data were collected as part of a wider study looking at current knowledge and best practice in IPC in the UK.^25^

Interviews used a structured schedule, designed to explore staff views and experiences of IPC interventions and practice (see supplementary material). This included examples of interventions identified from the IPC literature:

1. A multimodal toolkit including placing alcohol hand gel by bedsides, with supporting promotional materials, and a strategy to increase patient information
2. Strips of bright red tape on the corridor floor ending in an arrow pointing to the alcohol hand gel dispensers
3. Patients asking their health‐care workers if they had washed their hands

Staff were asked for their views about whether these would work in their own particular context. We also asked for their views on, and experiences of, IPC activity conducted on their ward, who should be involved in designing a new IPC intervention, and what made interventions work in practice. Interviews were conducted by LB and a second project researcher, either face‐to‐face, or by telephone if requested by the participant. Interviews lasted on average 25 minutes and were audio‐recorded and transcribed.

Data were analysed using the constant comparative method,^26^, ^27^ aided by the use of NVivo 11 software. Using the initial themes from a systematic literature review conducted as part of the wider study, a preliminary coding framework was developed. This framework was used to code the data, and was refined iteratively through close reading of the transcripts and data coding. The advantage of this approach to qualitative data analysis is the closeness to the “raw” data and the ability to generate meaning and theory from it iteratively.^28^ We explored themes around staff perceptions of patient and relative involvement in IPC, and barriers and challenges identified by staff in involving patients and relatives until data saturation was reached.

User views on the study as a whole were sought through a public consultation event facilitated by the research team in collaboration with Opinion Leader and the Patients Association, which included a sample of 15 carers and 26 patients recruited from across London. A multi‐stakeholder advisory board provided guidance throughout the study process. Members included: patients’ representatives, health‐care practitioners and experts in IPC, health systems, psychology, sociology and organizational analysis.

### 2.1 Ethics

Written informed consent was obtained prior to interview, and fully anonymized. Under the NHS Research Governance Framework, the study was constituted as a service evaluation and registered with each organization as such. Ethical approval was also granted by the University of Leicester's research ethics committee.

## 3 FINDINGS

Interviews were conducted with 35 frontline staff based in a range of clinical areas: intensive care; accident and emergency; and wards including elderly medicine, rehabilitation, gastroenterology and infection isolation. We also interviewed four members of senior management who were involved in implementing IPC interventions. Participants included medical directors, nurse consultants, matrons, ward managers, ward sisters, staff nurses, clinical support workers, student nurses, health‐care assistants and senior infection control nurses. We identified three broad themes: patient involvement as partnership or conflict; concerns about shifting responsibility for IPC; and patients as “risky bodies”.^29^, ^30^

### 3.1 Patient/relative involvement in IPC as partnership or conflict?

Staff expressed the view that involving patients and relatives in IPC was a good idea in principle. On the whole, staff were most supportive of approaches that involved patient co‐operation with self‐care practices, such as encouraging patient use of hand wipes. Sharing some responsibility with patients for aspects of IPC that were within patients’ own control was felt to help foster a partnership where patients and staff could work together to achieve goals of effective IPC. Staff engagement with this approach was reinforced when they saw that it was not disruptive, and that patients were compliant with the goal of reducing the risk of transmissible infections.

It needs everybody's participation I suppose, including the patients, because obviously if they're touching things and getting germs on their hands they're going to have to clean their hands as well. (Clinical Support worker 16)

The wipes … it's not an invasive thing and it's helping us with our infection control. (Clinical support worker 14)

Similarly, frontline staff had no qualms about promoting the use of hand gels, posters, ultraviolet technology and advertising campaigns to encourage both patients and their relatives to take IPC precautions. Staff regarded this as signifying the importance of cleanliness to the hospital as a whole, and as a valuable way of engaging both patients and relatives and visitors in the joint endeavour of reducing the spread of infection.

We've involved our visitors through articles in the local media, so we've covered hand washing. Periodically we'll then do a day where we'll stand in one of the main entrances and we'll stop visitors and we'll use the ultraviolet box…. To say how clean your hands are before and after you've washed them…raising awareness of the importance of hand washing. (Executive team member 4)

Staff had more mixed views about involving patients and relatives in co‐producing IPC through inviting them to be vigilant about staff IPC practices, such staff handwashing. Staff recognized that this potentially changed the nature of the relationship between staff and patients, and required health professionals to actively support and enable patients to speak up.

It's getting them to cross over really from their ‘Ooh she's a nurse, she knows what she's doing’ to ‘Well, actually, she might be a nurse but [that was bad] … and now she's coming to me with her hands.’ And it's just getting them to be confident enough to make that first step. (Nurse 6)

Staff expressed concerns that this type of involvement could be perceived as adversarial. Rather than enabling them to work in partnership with patients and their relatives to optimize IPC, this approach to involvement was seen as potentially disruptive to the staff‐patient relationship. Staff felt that patients and relatives could become anxious about having to take on this role of challenging staff, and they themselves were worried about how this made patients feel. Some staff also described their discomfort at the idea of being challenged by patients or their relatives. As a result, they felt less inclined to try to encourage this type of involvement in practice.

Some [patients] might be worried – in the back of their mind – that if they ask someone then it would become like an issue, and so you don't want that confrontation. (Nurse 20)

Because of it being so popularly talked about, even patients these days look at you going from one patient to another and they ask you have you washed your hands in between [… you] may be caught [out] by the patient and then you are embarrassed. (Nurse 10)

### 3.2 Patient/relative involvement and responsibility for IPC

Staff reported some concerns that efforts to involve patients in co‐producing IPC could mean shifting responsibility onto patients and relatives for reducing their own risks of infection as well as monitoring the IPC practices of staff. This was seen as creating problems in practice: tensions with negotiating patient co‐operation and patient autonomy; risk of obligating patients and undermining their trust; and ethical concerns about burdening patients and relatives.

### 3.3 Negotiating patient co‐operation

Although approaches that involved working co‐operatively with patients and relatives, including encouraging hand hygiene, were preferred in principle, these were dependent on patient and relative willingness to work together with staff towards IPC goals. While this was often unproblematic, staff did not *always* feel that their attempts to encourage patients and relatives to play a shared part in IPC were welcomed. In the case of providing patients with hand wipes, the aim was to make it as easy as possible for patients to clean their hands before eating and reduce the risk of infection, but staff sometimes found that patients were unwilling to co‐operate. They argued that patients did not always understand the rationale for being given hand wipes, and sometimes declined to use them. Where this happened, it conflicted with staff awareness of the very real (but invisible) threat of infection on a ward. This placed staff in a difficult position; they had to balance the patients’ decision not to use hand wipes with their own responsibility for managing infections. Staff could sometimes find it challenging to negotiate with patients to secure their co‐operation: having an open discussion about the patient role in managing infection risk seemed complicated by the recognition of the need to respect patient autonomy.

Some will say ‘my hands are not dirty’ and you have to say ‘I understand, but you have been laid in your bed all day and you have touched surfaces and it is for your own good.’ Some would still be adamant about it. (Student nurse 13)

We have those little wipes and before [meals] we're like ‘everyone needs to wash their hands!’ But obviously it's patients’ choice to; some patients won't like doing that. (Student nurse 17)

### 3.4 Responsibility for IPC: obligating patients

Some staff questioned whether it was right to assign responsibility to patients and relatives for monitoring and speaking up about staff practices. Nurses in particular tended to express the view that while patients and relatives should feel welcome to ask about hand hygiene and IPC procedures, it was their professional responsibility to keep patients safe, and that obligations should not be placed on the individual patient or their relatives. Revealing invisible risks of infection to patients who may be unaware of those risks, or not cognizant to deal with them, was seen as having the potential to undermine trust and threaten the relationships between staff and patient.

Personally, I'm not a huge fan of this… what we try and engender in healthcare is a level of trust between clinician and patient… it's almost unpicking that level of trust… [When] you start down that path, it's where do you stop? (Executive team member 1)

I think it is difficult for patients, particularly when nurses are looking after them, I think sometimes it can be difficult for them to say, ‘By the way, have you washed your hands?’ Because they do trust people, and they do feel it might be an impingement, asking nurses if they have done that. (Ward manager 6)

### 3.5 Ethical concerns about burdening patients and relatives

Further to the concerns about undermining trust, some staff also expressed concerns about the ethics of placing an additional burden of responsibility on patients and relatives by expecting them to participate actively in IPC efforts. Staff argued that patients and relatives could not always be expected to play an active role in co‐producing IPC: patients were often very sick or in pain, and relatives were often distressed and overwhelmed by the experience of their loved one being acutely or seriously unwell in hospital. The context of the ward had an impact: in the ICU, and similarly in other settings when a patient was acutely ill, asking relatives and visitors to challenge health‐care workers could at times be seen as unethical or inappropriate.

We had badges encouraging the staff, and we had signs encouraging the patients to ask. I think it's a good idea, in theory, but… it has limitations… The majority of our patients, it's an acute admission, they're in pain, they're in distress, they're in discomfort. (Senior charge nurse 39)

Obviously people's relatives can be quite distraught in that situation. To be honest it's probably the last thing they're thinking about. (Nurse 38)

### 3.6 Patients as risky bodies: lack of patient ability or capacity as a barrier to involvement in the co‐production of IPC

Staff recognized that patients did not always have the ability or capacity to be involved as partners in IPC. Caring for patients with dementia or confusion was seen as raising particular challenges in relation to working together with patients to achieve IPC goals. These patients with reduced capacity were seen as unable to play an active role in IPC, and, in fact, staff described how patients who lacked capacity could instead create risks and act as threats to IPC efforts that had to be managed. For example, patients sometimes wandered in and out of isolation areas as nurses could not always monitor their movements within wards.

I know one of [my patients] kept walking up and down the corridor, and it's hard to explain to dementia patients—you're contagious, you need to stay in your room. (Student nurse 30)

Staff also revealed the tensions in ensuring a safe, infection‐controlled environment for dementia patients. On discussing the type of intervention that would promote IPC, such as additional signage, they expressed concerns that this would add to the confusion that patients with dementia might experience.

People with dementia, you need a clear environment. You don't want clutter, it needs to be very stark almost, and you would have bare surfaces….So that the red tape along the corridor, along with things like, you know, printed footprint signs at the door which says ‘Wash your hands when you go in here’, plus a whole range of notices on the door saying ‘Visiting time’ and, that's very bad for people with dementia. (Site 1, chief nurse)

Nursing staff also noted that sometimes patients presented risks that ran counter to the requirements of IPC policies and practice, such as the risk that patients with confusion or experiencing alcohol withdrawal would drink alcohol hand gel, and that providing a safe environment required considering other risks alongside the risks of infection. This meant they had to carefully consider patient needs and vulnerabilities as well as recommended IPC procedures.

We occasionally have a problem with the hand gel, because some of our patients [have alcohol problems] you know, and it's alcohol based, isn't it?… They have drank it on several occasions. (Sister 22)

In the accounts of staff, it was clear that for some patients, optimizing IPC was about findings ways to work around the challenges presented by “risky bodies”,^29^, ^30^ rather than working in partnership with patients and involving them actively in co‐producing good IPC practice. There seemed to be no easy solutions to these problems and no evidence from the literature, with nurses working on a case‐by‐case basis to keep patients as safe as possible.

## 4 DISCUSSION

### 4.1 Summary of findings

This study aimed to understand staff views of patients and relative involvement in aspects of IPC. Approaches founded in encouraging co‐operation and compliance, as opposed to invoking conflict and challenge, were seen as more acceptable and effective ways of involving patients and relatives in the co‐production of IPC. Staff could, however, sometimes find it difficult to secure compliance to IPC practices including the use of wipes and handwashing from patients and relatives, and were unsure how to tread the line between respecting individuals’ autonomy, and performing their duty to protect patients from the risk of infection. Staff were concerned that asking patients and relatives to speak up about IPC placed too much obligation on patients and relatives to monitor safety, and could undermine trust and threaten the relationships between staff and patient.

### 4.2 Strengths and limitations

Our study highlights the tensions evident in the operationalizing the aspiration of IPC as “everybody's business” through involving patients and relatives in the co‐production of safer health care: between the moral imperative to empower patients as partners in their health care,^31^ and the risk of over‐burdening vulnerable patients and shifting responsibility from the professional onto the patient.^16^, ^17^, ^20^

Our study is limited in that it included only two hospital sites, but participants were sampled to ensure a range of job roles and experience. The discussion was focused on potential interventions, but the majority of staff had experienced one of more of the interventions in practice, and were also able to discuss other approaches to involving patients and relatives in IPC that they had experienced.

### 4.3 Comparison with previous literature and implications for practice

Co‐production models suggest that rather than shifting the onus from the professional onto the patient for ensuring safety,^32^ there is a need to establish shared goals as a first step to working together, and for explicit sharing, rather than handing over, of responsibility. The very nature of IPC, however, makes patient involvement in it difficult. Patients in hospital settings expect to be given care to help them to recover or their condition to improve, and do not necessarily anticipate being harmed from invisible risks in the very environment in which they are receiving care. Staff recognize that patients, therefore, may not perceive the ward environment to be their responsibility. This sits in contrast to other areas of patient involvement in patient safety. For example, patient involvement in medication safety may be more straightforward because many patients are familiar with, and already hold some responsibility for, taking their own medications. They thus retain some ownership over this tangible issue, which is something within their control. As Batalden et al note

It is neither possible nor desirable to share power and responsibility equitably between patients and professionals in all situations. The burden of responsibility for medical and surgical error, for example, must fall disproportionately on healthcare professionals.^3^

Staff felt that patients and relatives were sometimes willing to take on some responsibility for IPC, but that the ability to be involved could change over time depending on circumstances. This indicates that a more nuanced approach to involvement may be required. Patient and relative capacity and willingness to be involved in IPC should be seen as context specific and fluctuating,^33^ and in a similar way to arguments that have been made for consent to research, staff may need to re‐negotiate the level and nature of involvement as circumstances change.^34^

Staff also recognized that regulating and controlling “risky bodies”,^30^, ^35^ where patients who lacked capacity created risks, sat in tension with involvement. It was a difficult matter to involve patients in reducing the spread of infections if they had delirium, dementia or confusion or “roamed” the wards. Staff felt unsure how to go about negotiating with patients and prioritizing risks to ensure that patients were safe, especially when the need to manage other risks competed with IPC practice. In line with Batalden, our study reveals how the system, or environment in this case, constrains the effective partnership between patient and professional.^3^ Our study suggests that staff need skills in navigating the line between collaborative and paternalistic approaches and behaviours, with a recognition that paternalistic approaches may sometimes be necessary in IPC, particularly when patients have diminished capacity or their actions put others at risk, despite the increased recognition of the patient's right to autonomy.^36^ Staff may also need access to training, support and guidance on producing “good enough” IPC in ways that take into account individual patient circumstances.

We studied the involvement of patients and relatives in the co‐production of IPC in the context of care delivery: effective approaches to co‐production of IPC are likely to require strategies that lie beyond the point of care. One way to address these issues may be to collaborate either earlier or later in the care process and/or to use different techniques, to enable a dialogue without consequences between health‐care professional and patients. For example, Wyer et al show how video reflexive technology, when used with patients and clinicians together, enabled clinicians to reflect on their IPC practices. Patients felt able to raise issues “through a third party” and reduced the potential for patients and staff to deal with confrontational moments; “brokering new relationships between them”.^37^ There is value in involving patients and relatives beyond the point of care, either during the intervention design stage or following discharge to support collaborative working in IPC. This would help staff to optimize their IPC practice when patients lack capacity or present IPC risks. Our study highlights the need for further research in this area.

## 5 CONCLUSIONS

Efforts to achieve genuine and appropriate patient and relative involvement need to take into account staff motivation to collaborate with patients and relatives in optimizing IPC, and the barriers they see in involving patients in IPC. Our work describes staff views on the challenges of involving patients in co‐producing IPC as part of care delivery. Establishing how and when to engage patients and relatives to play a role in co‐producing IPC at the point of care can be complex, and our work highlights the need to explore how staff can be supported to make ethical and appropriate decisions about involving patients and relatives that address individual characteristics and needs. Staff responsibility for protecting patients from the risk of infection may sometimes need to take priority over prerogatives to involve patients in the co‐production of IPC.

## CONFLICT OF INTEREST

The authors declare no conflicts of interest with respect to the authorship or the publication of this article.
