# Supplementary material for: Making infection prevention and control everyone's business? Hospital staff views on patient involvement
Source: Health Expect. 2019 Feb 17;22(4):650–6. doi: 10.1111/hex.12874 (PMC6737752; doi:10.1111/hex.12874)
Supplement: Supplementary file 1 [file HEX-22-650-s001.docx]

## Case studies - interview guide

*The following questions need not be covered in this particular order but rather the interview should flow as freely and naturally as possible. The interviewer will prompt as appropriate with phrases such as ‘can you tell me a little more about that’, ‘can you give me an example of that’, etc*

**Welcome and introduction - Seek consent to continue and to audio-record the interview.**

**Re-cap of project and plan for interview**

*The interview will explore: your views on, and your experiences of implementing infection control strategies. The aim is to understand how hospital staff would get on in implementing examples of strategies identified as effective by a systematic review of literature.*

*All questions apply to your trust, and we are interested in your perception, on the basis of knowledge and experience. If you do not feel you are able to comment please say so.*

*Do you have any questions before we start?*

1. **Project administration**

| Researcher: | Date: |
| --- | --- |
| Email: | Telephone: |
| Consent form attached | Participant Info provided |

1. **Respondent profile**

- Organisation name and type of organisation (if not previously established)-
- What is your present job title?
- How long have you been in this position?

1. **Infection Prevention and Control in the Trust**

| How is infection prevention and control perceived within your Trust?  Is IPC among the top priorities for your organisation?   - If so, how is that demonstrated in practice? |
| --- |
| What sources of pressure are there for your trust to improve its IPC performance? |
| Can you give me an overview of what IPC activity there is in your ward/ clinical area? |

1. **Views on "promising" interventions**

| Our literature review hints to XYZ as a promising intervention. (Provide additional details of the intervention ― see section 6)   - Has anything similar been introduced in your trust? - If not, how would you feel if this intervention was introduced within your trust? |
| --- |
| What would help/ has helped you to introduce an intervention like this?  Prompts: training, support, structural, cultural, who involved, cost? |
| Did you encounter/ can you anticipate potential barriers and challenges? |
| Who needs to be involved with designing an intervention like this to make sure it is successful? (prompts: nursing staff, healthcare assistants, patients, consultants)  Who needs to be involved when it’s implemented to make it a success? |

1. **Real experience with "promising" and "challenging" interventions**

| From your experience, can you identify a particular IPC intervention that you deem particularly promising in practice? |
| --- |
| Why would you consider this successful? |
| What helped to make this successful?  Prompts: time taken to implement, training, support, structural, cultural, who involved, cost |
| Similarly, from your experience, can you identify a particular IPC intervention that you deem particularly challenging (e.g. that was rejected or discontinued in the Trust)? |
| Why do you think this didn’t work? |
| Based on your experience, what are the key features that an IPC intervention should possess in order to be successful?  Prompts: think about the whole life cycle of an intervention, including planning and design, implementation, post-implementation (when the "new" becomes the "norm") |
| What do you think are the challenges of scaling-up interventions that originally targeted a single ward or a single professional group?  How do we make changes sustainable in practice? |

**Anything not covered?**

Is there anything that we haven’t covered in the interview that you think we should know or think about?

**Closing and thanks**

Check that the participant is still happy for us to use all the information provided. Thank for their time and contribution.

1. **"Promising" interventions identified with the literature search**

| Description of intervention: Toolkit that included placing alcohol hand rub beside patients, along with posters and supporting marketing materials, initiation of a guide to implementation and of a strategy aimed at increasing patient information and empowerment.  Key message of the study: Multimodal strategies are effective in improving hand hygiene compliance because of a change in the way that staff perceived the practice of hand hygiene from low priority to a core element of daily practice. |
| --- |
| Description of intervention: Strip of bright red tape along the corridor on the approach leading to the ward entrance, producing an arrow that pointed to the two alcohol gel dispensers on the wall.  Key message of the study: Strategies to improve hand hygiene compliance may place inexpensive, visible signs on the walls to remind healthcare workers about hand hygiene, but may need to be expanded by broader culture change programme to sustain improvements. |
| Description of intervention: Patients asking their healthcare workers if they had washed their hands.  Key message of the study: Strategies that allow patient to provide feedback/reminders to healthcare workers are effective, empower patients with responsibility for their care, provide staff with a continuing means for providing handwashing education without additional staff, and can save costs for a hospital; they can be effectively anticipated by administrative support to the initiative through flyers and letters. |
| Key messages from other non-intervention studies:   1. Strategies to change behaviour need to present hand hygiene as a learning process and need to consider the cognitive process of reflection and self-assessment, so that student nurses can better understand their needs, seek for proper information, and change behaviour. 2. Effective strategies to change behaviour need to first understand a professional group's habitus. 3. Strategies to change behaviour need to take into account processes of socialisation and "fitting in" to the team. 4. Active participation in educational activities is more conductive to behavioural change, so practice development (an activity that aims to change practice and improve patient care) should underpin any changes. Assertiveness training should be included in pre-registration nursing training to equip students with the skills to challenge clinical practices and effective mentorship can increase the students' ability to fit into clinical settings. 5. Strategies to improve hand decontamination should emphasise hand hygiene responsibility at key point during nurse training and hand hygiene skills should be assessed at university every year during training. 6. Nurses perceive MRSA to be out of control and they are not overly concerned about its management -- mainly because of workload and busy schedules. Successful strategies to change behaviour: 1. need to use education and increase clinical awareness by infection control teams, supported by the appropriate level of nursing management; 2. require strong leadership and clear direction; 3. require that infection control teams engage with staff more frequently. |
